# Transcriptomes and pathways associated with infectivity, survival and immunogenicity in *Brugia malayi *L3

**DOI:** 10.1186/1471-2164-10-267

**Published:** 2009-06-15

**Authors:** Ben-Wen Li, Amy C Rush, Makedonka Mitreva, Yong Yin, David Spiro, Elodie Ghedin, Gary J Weil

**Affiliations:** 1 Department of internal medicine, Washington University School of Medicine, St. Louis, MO 63110, USA; 2 The Genome Sequencing Center, Department of Genetics, Washington University School of Medicine, St. Louis, MO 63110, USA; 3 J. Craig Venter Institute, 9704 Medical Center Drive, Rockville, MD 20850, USA; 4 Divisions of Infectious Diseases, University of Pittsburgh School of Medicine, Pittsburgh, PA 15261, USA

## Abstract

**Background:**

Filarial nematode parasites cause serious diseases such as elephantiasis and river blindness in humans, and heartworm infections in dogs. Third stage filarial larvae (L3) are a critical stage in the life cycle of filarial parasites, because this is the stage that is transmitted by arthropod vectors to initiate infections in mammals. Improved understanding of molecular mechanisms associated with this transition may provide important leads for development of new therapies and vaccines to prevent filarial infections. This study explores changes in gene expression associated with the transition of *Brugia malayi *third stage larvae (BmL3) from mosquitoes into mammalian hosts and how these changes are affected by radiation. Radiation effects are especially interesting because irradiated L3 induce partial immunity to filarial infections. The underlying molecular mechanisms responsible for the efficacy of such vaccines are unkown.

**Results:**

Expression profiles were obtained using a new filarial microarray with 18, 104 64-mer elements. 771 genes were identified as differentially expressed in two-way comparative analyses of the three L3 types. 353 genes were up-regulated in mosquito L3 (L3i) relative to cultured L3 (L3c). These genes are important for establishment of filarial infections in mammalian hosts. Other genes were up-regulated in L3c relative to L3i (234) or irradiated L3 (L3ir) (22). These culture-induced transcripts include key molecules required for growth and development. 165 genes were up-regulated in L3ir relative to L3c; these genes encode highly immunogenic proteins and proteins involved in radiation repair. L3ir and L3i have similar transcription profiles for genes that encode highly immunogenic proteins, antioxidants and cuticle components.

**Conclusion:**

Changes in gene expression that normally occur during culture under conditions that support L3 development and molting are prevented or delayed by radiation. This may explain the enhanced immunogenicity of L3ir. Gene Ontology and KEGG analyses revealed altered pathways between L3 types. Energy and "immune pathways" are up-regulated and may be needed for L3i invasion and survival, while growth and development are priorities for L3c. This study has improved our understanding of molecules involved in parasite invasion and immune evasion, potential targets of protective immunity, and molecules required for parasite growth and development.

## Background

Filarial nematodes cause a number of serious diseases in humans (e.g., lymphatic filariasis and onchocerciasis) and animals (e.g., canine heartworm disease). Filarial parasites have complex life cycles that share the following general features: Arthropod vectors (ticks, mites, mosquitoes, black flies) ingest microfilariae (Mf) that circulate in the blood or live in the skin of definitive vertebrate hosts. Ingested Mf penetrate the arthropod midgut and develop over a period of 10 to 14 days to become third-stage infective larvae (L3). Filarial L3, developmentally arrested in arthropod vectors, resume development after they are transferred to a permissive vertebrate host. The L3 is the critical life stage for infection, as it is the transitional stage between the vector and the mammalian host. Molecular mechanisms associated with this transition are poorly understood; they may provide important clues for new therapies and vaccines to prevent filarial infections.

Prior studies have used different methods to investigate adaptations that occur in filarial L3 after they enter mammalian hosts (or in response to culture conditions that mimic the mammalian environment). Early studies used metabolic labeling and surface labeling techniques with SDS-PAGE to detect altered protein patterns [1-3]. Molecular biological approaches have included differential hybridization [2] and analysis of expressed sequence tags (ESTs) from cDNA libraries from pre- and post-infective L3 [4,5]. These studies provided interesting, preliminary information regarding adaptations associated with the transition of L3 from arthropod to mammalian hosts.

In addition to being the infective stage for vertebrate hosts, L3 are also important targets of protective immunity [6-8]. A number of studies have shown that irradiated L3 (L3ir) are more effective for inducing immunity to infection than L3 freshly isolated from vectors (L3i) [9-11]. Potential targets of such protective immunity have been identified by screening crude antigen preparations and expression libraries with sera from animals vaccinated with irradiated L3s [11,12]. We have previously used quantitative real-time PCR (qRT-PCR) analysis to identify a limited number of genes that were differentially expressed by cultured or irradiated L3 [5]. The present study builds on this prior work to more broadly describe changes in L3 gene expression associated with the transition from arthropod to mammalian host conditions (presumably related to invasion, growth, and immune evasion) and effects of radiation on gene expression (which may provide insight into targets of protective immunity). While prior studies focused on ESTs from cDNA libraries, the current study benefited from recent advances in filarial genomics [13] and microarray technology to provide more complete expression profiles. We have also compared changes in filarial L3 gene expression associated with the vector-vertebrate transition with changes associated with the soil-vertebrate transition in *Ancylostoma caninum *(canine hookworm) and with dauer exit in *Caenorhabditis elegans*.

## Results and discussion

### General properties of the *B. malayi *Version 2 array (BmV2 array)

The oligonucleotide elements on the BmV2 array represent ~86% of currently annotated *B. malayi *gene models defined by Ghedin et al [13]; 11,975 of 15,412 *B. malayi *sequences correspond to 9,798 unique annotated gene models, and the remaining 3,437 sequences that do not match annotated gene models represent genes that have not yet been sequenced or annotated in the *Brugia *genomic DNA. The BmV2array elements included all genes in the *Wolbachia *genome and ESTs from *O. volvulus *and *W. bancrofti *with little homology to known *B. malayi *sequences. A homology search of the 18,104 sequences from which the oligos were derived vs. the NCBI non-redundancy (NR) database revealed that ~69% (12, 423/18,104) had significant homology to publicly available known or unknown proteins from other species (e-05) [See additional file 1]. Of 15,412 *Brugia *sequences, 9,824 sequences had best matches to 8,038 unique *C. elegans *genes with RNAi information; 37% of these genes had RNAi phenotypes [See additional file 2].

Functional assignments for elements on the microarray were performed using InterPro, KEGG, and GO analysis. The complete results are reported in supplemental materials [protein domain matches in additional file 3; pathways in additional file 4]. The top 20 most abundant protein domain matches in the oligoarray are presented in additional file 5. Based on InterPro protein domain matches, 48% of the products of genes on the array mapped to one or more gene ontology terms [14,15]. These GO associations are available online through the Amigo viewer 

### Overview of differences in gene expression by L3 type

**Figure 1:**
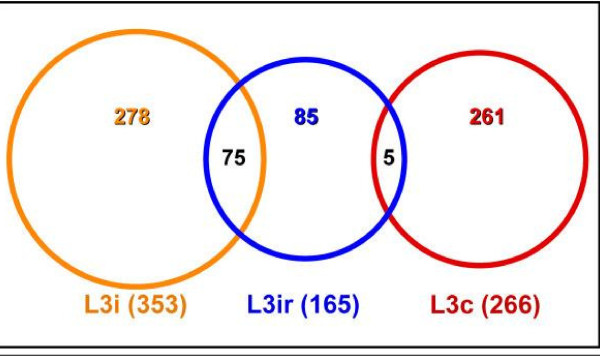
**Venn diagram show distribution of up-regulated genes in three L3 type**. For example, out of 353 L3i up-regulated genes, 75 genes are up-regulated in both L3i and L3ir relative to L3c.

A complete list of elements for the array with oligonucleotide sequences and hybridization data are available online through the link: . The expression profile analysis included elements with hybridization signals above background for at least one condition in pair-wise comparisons (L3i *versus *L3c and L3ir *versus *L3c). A total of 774 genes exhibited significant differential expression in pair-wise comparisons (≥ 2 fold differences with *P*-values < 0.01) [See additional file 6]. 353 of these genes (~46%) were up-regulated in L3i relative to L3c (the "L3i gene set"); 266 (~33%) were up-regulated in L3c (the "L3c gene set", with 244 genes up relative to L3i and 22 up relative to L3ir); 165 (~21.3%) were L3ir-up-regulated relative to L3c (the "L3ir gene set') (Figure 1). The L3i gene set contains genes required for L3 survival in mosquitoes and for infection of mammalian hosts. The L3c gene set represents genes induced by culture conditions that mimic the mammalian environment, and the L3ir gene set represents genes induced by irradiation. Interestingly, some genes exhibited up-regulated expression in more than one condition (Figure 1).

We were surprised to see that the L3i list was the largest of the three L3 gene sets. However, this was consistent with the finding that more *Ancylostoma canium *L3 (AcL3) genes had reduced transcription after serum stimulation (equivalent to our L3c) relative to non-serum stimulated AcL3 (equivalent to our L3i) [16,17]. This result suggests that the L3i is not simply a quiescent or resting stage. L3i are actively preparing for their transition into the mammalian host by expressing proteins that are needed for processes like invasion and immune evasion and proteins for survival (stress resistance and antioxidants).

As expected, irradiation repressed gene expression globally; the L3ir gene set was the smallest of the three L3 gene sets. Of 165 L3ir-up-regulated genes, 75 (45%) genes were also up-regulated in L3i relative to L3c (Figure 1). Most of these genes (54/75) were expressed at higher levels in L3i than in L3ir, and only 4 (intermediate filament, collagen family, and Gly-rich proteins) had higher expression in L3ir; 17 were expressed at similar levels in both conditions [See additional file 6].

We previously reported partial conservation of L3i expression patterns in L3ir based on an EST analysis with qRT-PCR confirmation data [5]. The current analysis has confirmed and extended this observation. Several collagen genes and other so-called immunogenic genes (*Gln-rich*, *spx-1*), were over-expressed in L3ir relative to L3c [See additional file 6]. Persistent expression of important targets of protective immunity may explain why L3ir are better inducers of anti-L3 immunity than L3i, which are likely to change gene expression to resemble L3c with reduced expression of these proteins shortly after infection.

The taxonomic distribution of proteins encoded by the up-regulated gene models in each condition are shown in Table 1. More proteins encoded by *B. malayi *gene models in the L3i and L3ir gene sets are nematode-specific as defined by Ghedin et al [13] compared to those in the L3c gene set. Nematode-specific gene products are attractive candidate targets for new drugs and vaccines because they lack homology to mammalian proteins.

**Table 1:** Taxonomic distribution of known proteins encoded by up-regulated gene models in the L3 gene set

**Gene set**	**Only filarial**	**Other nematodes**	**Nematode-specific**	**Others**	**No. gene models**
L3i	13	64	77 (37%)	132	209
L3ir	11	34	45 (48%)	49	94
L3c	9	21	30 (25%)	92	122
Total	33	119	152	273	425

RNAi information for *C. elegans *homologues of genes associated with different BmL3 types are listed in additional file 7. RNAi resulted in phenotypes in 10%–14% of all *C. elegans *predicted genes [18-20] and in 27% of genes with evidence of expression [21]. In contrast, a much higher percentage of *C. elegans *genes with strong homology to genes associated with different BmL3 types had RNAi phenotypes (Table 2). *C. elegans *genes homologous to genes up-regulated in BmL3c were more likely to have RNAi phenotypes (60%) than those with homologues upregulated in L3i or L3ir. This was consistent with results reported for *Strongyloides stercoralis *[22]. The most common RNAi phenotypes observed for *C. elegans *genes with homology to genes in the BmL3 gene sets were developmental delay (slow growth, larval arrest and lethal), embryonic lethal, and sterile [See additional file 7].

**Table 2:** RNAi phenotypes for differentially expressed filarial genes with close *C. elegans *homolog

**Gene set**	**total matches**	**Unique matches with RNAi information**	**No. with RNAi phenotypes (%)**
L3i	182	137	58 (42%)
L3ir	100	77	29 (38%)
L3c	117	91	55 (60%)
Total on the array*	9824	8038	3011 (37%)

* Elements with homologs in *C. elegans *(1E-05 or better)

### Distinct functions of known genes associated with different L3 gene sets

The L3c gene set had a higher percentage of novel genes (42%) than the L3ir (27%) and L3i (28%) gene sets (Table 3). This is consistent with results from our study of ESTs from BmL3 cDNA libraries [5] and a recent study of transcriptional changes in AcL3 [17]. These novel genes may be filaria-specific, because they have no similarity to known genes in public databases. Additional work will be needed to determine the function of these genes. In the meantime, we can learn a lot from analysis of differentially expressed known genes. We grouped the most abundant up-regulated known genes in each L3 gene set by biological function (Table 4, 5, 6), and this information is discussed below.

**Table 3:** Distribution of BLAST matches in the L3 gene set

**Gene set**	**Unknown**	**Known**	**Total**
L3c	113 (42%)	154 (58%)	266
L3i	104 (28%)	249 (72%)	353
L3ir	44 (27%)	121 (73%)	165

Note: Blast run against NCBI NR database, cut off 1e-05

**Table 4:** Major known genes by functional class in the L3i gene set

**Functional class**	**Gene**	**Oligo ID**	**Fold**	**NCBI accession number and description**
***Immunomodulators***	*alt-1*	BMC00123	*35*	AAB03902.2| Av18, Alt 1 variant
	*alt-2 *(2)	14863.m00021	*20*	AAF01224.1| Alt2 variant
	*cpi-1 *(3)	BMC12220	*41*	AAD51085.1| cystatin-type cysteine proteinase inhibitor CPI-1
	*cpi-2 *(2)	BMW01194	*4*	AAD51086.1| cystatin-type cysteine proteinase inhibitor CPI-2
	*spn-1 *(5)	AA840996	20	AAB42377.1| BmSERPIN
***Anti-oxidants***	*gst *(1)	BMC02401	2.6	CAA73325.1| glutathione transferase
***detoxification***	*sod *(3)	BMC00677	2.2	AAR06638.1| superoxide dismutase
	*akr *(1)	AA841533	15.4	NP_509242.1| (Aldo/keto reductase family proteins)
	*grx *(1)	13358.m00042	4.2	CAE74325.1| Glutaredoxin-like region
	*tpx-1 *(1)	TC2998	11.2	AAN34969.1| thioredoxin 1; ov-thioredoxin 1
	*tpx-1 (*3)	BMC12191	2.4	AAL91107.1| thioredoxin
	*tpx-2 *(1)	BMC12241	3.7	Q17172|TDX2hioredoxin peroxidase 2
	*trxR *(1)	14972.m07078	4.3	AAD46625.1| thioredoxin reductase homolog
	*pxn-2 *(1)	BMC12240	4.3	AAC77922.1| peroxidoxin-2
***Proteases***	*cpl-1 (*7)	BMC01348	10.7	AAK16513.1|cathepsin L-like cysteine proteinase
	*cpl-4 (*4)	BMC06080	20.8	AAT07057.1| cathepsin L-like cysteine proteinase
	*cpl-5 *(2)	BMC11984	19.7	AAT07058.1| cathepsin L-like cysteine proteinase
	*mtp *(1)	BMC10914	4.6	BAC66058.1| matrix metalloproteinase
***Pathogenesis***	*val-1 *(7)	BMC12291	10	gb|AAB97283.2| vespid allergen antigen homolog
***related protein***	*nlt-1*	BMC12455	7.5	AAD11970.1| 24 kDa secreted protein
***Parasitism***	*ov103*	12584.m00062	13.6	AAA63412.2| microfilariae surface-associated
		BMC01088	3.2	AAF76925.1|1 hypothetical esophageal gland cell secretory protein 11
***Stress resistant***	*shsp*	TC3642	10.2	AAB07020.1| small heat shock protein
***Cuticular components***	*elo-3*	14954.m01636	58.2	ref|NP_501147.2| fatty acid ELOngation family member (elo-3)
***Related member***	*col *(47)	TC2764	11.2	NP_500520.1| COLlagen family member (col-34)
	*Grl-4*	14083.m00057	8.9	NP_501166.2| GRound-Like (grd related) family member (grl-4)
***Highly immunogenic***	*Val (8)*	BMC12291	9.9	aaB97283.2 vespid allergen antigen homolog
	(3)	WB-contig_1280	10.8	AAA63412.2| microfilariae surface-associated
	(1)	BMC07029	2.8	P23730|IFEA_ASCSU Intermediate filament protein A
	(3)	14323.m00082	7.1	AAN62757.1| larval allergen [Brugia malayi]
	*spx-1 (4)*	BMC05831	6.1	AAA27864.1| SPX-1
	*(2)*	BMC02375	3.6	AAC48290.1| Gln-rich protein

Numbers in parentheses represent the number of oligos that match a particular gene that was upregulated.

**Table 5:** Major known genes by functional class in the L3ir gene set

**Functional class**	**Gene**	**Oligo ID**	**Fold**	**NCBI accession number and description**
***Irradiation responsive***				
	*aid *(3)	BMC04376	9.2	CAA62521.1| cytidine deaminase [Brugia pahangi]
	*ada *(1)	BMC12015	3	NP_501087.1| C06G3.5a
	*up ase *(1)	13555.m00076	11.2	NP_498671.2| ZK783.2 (Uridine phosphorylase)
	zinc finger protein (1)	BMC11725	2.2	CAA34357.1| zinc finger protein
	Gated Ion channel (1)	AI856833	5.5	NP_001023062.1| C43F9.9 (Neurotransmitter-gated ion-channel)
	*aqp *(2)	BMC02613	2.7	NP_502044.1| AQuaPorin or aquaglyceroporin related family member (aqp-3)
	Transketolase (1)	BMC06090	3.8	NP_501878.1| F01G10.1 (Transketolase)
***Cuticular components Secretory and membrane proteins Immunogenic***				
	*col *(64)	BMC03733	4	NP_001040924.1| COLlagen family member (col-14)
		13970.m00031	3	YP_293890.1| putative membrane protein
		BMC02058	2.2	NP_500485.1| F49F1.1 (Secreted surface protein)
	Gln-rich (3)	BMC11922	5.9	AAC48290.1| Gln-rich protein
	Oveg 1 (2)	BMC05959	4.3	AAB35895.1| Oveg1
	Juv-p120 (1)	14672.m00017	2.2	AAS92593.1| excretory/secretory protein Juv-p120
	*bmif *(1)	BMC07029	3.1	P23730|IFEA_ASCSU Intermediate filament protein A
	*spx-1 *(3)	BMC06144	2.3	AAA27864.1| SPX-1
**Antioxidant detoxification**				
	*pxn-2 *(1)	BMC12240	3	AAC77922.1| peroxidoxin-2
	*tpx-2 *(1)	BMC12241	2.8	Q17172|TDX2_BRUMA Thioredoxin peroxidase 2
	*gst *(1)	BMC02401	2.3	CAA73325.1| glutathione transferase
	*tpx-1 *(1)	TC2998	3.6	AAN34969.1| thioredoxin 1; ov-thioredoxin 1
**Fatty acid binding**				
	*scp *(1)	TC3583	3.6	XP_392432.2| similar to Sterol carrier protein X

Numbers in parentheses represent the number of oligos that match a particular gene that was upregulated.

**Table 6:** Major known genes by functional class in the L3c gene set

**Function class**	**Gene**	**oligo ID**	**Fold**	**NCBI accession number and description**
***protein synthesis***				
	rRNA promoter binding (1)	BMC10335	4.6	NP_671477.1| rRNA promoter binding protein
	RNA binding (3)	BMC04564	2.5	AAC47624.1| putative RNA binding protein
	Elongation Factor (5)	AA842168	4.7	CAB40840.1| elongation factor 1 beta
	*rps or rpl *((38)	BMC00849	4.6	AAN05602.1| ribosomal protein S7
	Zinc finger (8)	14287.m00022	9.7	P_698623.1| PREDICTED: similar to zinc finger
	*hsp*(1)	13293.m00125	9.5	AAY66912.1| putative heat shock-related protein
	*Pin1 *(2)	AA109462	6.5	AAD01597.1| peptidyl-prolyl cis-trans isomerase
***cell cycle***				
	*mab-21 *(1)	BMC02725	4.5	NP_497940.2| Male ABnormal family member (mab-21)
	*tctp (Tph) *(1)	BMC12076	2.9	AAK71499.1| translationally controlled tumor protein-like protein
	Histone (7)	12698.m00329	13.5	P30757|H2B_SIPNU Histone H2B
	Actin (1)	BMC06331	6	AAU94673.1| actin
	Tubulin (3)	BMC01602	3.2	AAA27865.1| beta-tubulin
***fiber growth***				
	MSP fiber proteins (MFP) (2)	BMC01338	6.9	AAP94887.1| MFP3
		BMC02125	3.7	AAP94885.1| MFP1-beta
***development cell phagocytosis***	*sHSP *(2)	15334.m00009	4.7	CAA61152.1| small heat shock protein
	*ced-2 *(1)	14972.m07627	7.8	NP_500105.1| CEll Death abnormality family member (ced-2)

Numbers in parentheses represent the number of oligos that match a particular gene that was upregulated.

### The L3i gene set contains genes associated with immune evasion, stress resistance and infectivity

Several classes of known genes were highly expressed in the L3i gene set as shown in Table 4. Immunomodulatory genes are important for invasion and establishment of infection in mammalian hosts [23], and prior studies have suggested that L3i modulate the innate immune system [6]. Microarray experiments showed that live BmL3 induced functional alterations of epidermal Langerhans cells (LC) and diminished their ability to present antigens to T-cells. Thus, L3 may actively suppress host immune responses to the parasite immediately after invasion [24]. Our findings suggest a molecular basis for such immune evasion, because a number of L3i up-regulated genes encode immune-modulators. These molecules may suppress local immunity to promote parasite survival just after invasion. For example, filarial ALT proteins encoded by the *Bm-alt *gene family modulate cytokine-induced signaling in immune cells and may render incoming L3 more resistant to IFN-γ induced killing by macrophages, as shown in a transgenic *L. mexicana *system [25]. Filarial cystatins are well-described pathogenicity factors that down-regulate T-cell proliferation and induce anti-inflammatory cytokine responses [26,27]. The cystatin protein encoded by *Bm-cpi-2 *interferes with antigen presentation by inhibiting multiple cysteine protease activities including the key enzyme asparaginyl endopeptidase [28]. *Bm-spn-2*, a member of the *B. malayi *serpin family, has been reported to impair granulocyte function by inhibiting two neutrophil enzymes, cathepsin G and neutral elastase [29].

The L3i gene list includes a broad spectrum of anti-oxidant and detoxification gene families such as superoxide dismutase (*Bm*-s*ods*) [30], thioredoxin peroxidase *(Bm-tpx-2) *[31] glutathione S-transferase (*Bm-gst*) [32,33], and aldo/keto reductase family proteins [34]. Since non-feeding filarial L3 are protected by a resistant cuticle, increased transcriptional activity of these genes may be needed for detoxifying endogenous compounds such as metabolites of fatty acids [35]. Fatty acids appear to be an important energy source in L3i as shown below. These enzymes also protect L3 from reactive oxygen species (ROS) generated in energy production process (oxidative phosphorylation) [36]. In addition, L3i are preparing to cope with oxidative stress upon host entry. Prior studies have shown that filarial larvae (L1 and L3) are more susceptible to killing than adult worms via antibody-dependent cellular cytotoxicity *in vitro*. This killing is believed to depend on reactive oxygen and nitrogen intermediates produced by activated myeloid cells [37,38] L3 must be able to resist oxidative stress to survive in mammalian hosts.

The L3i gene set also includes several proteases that may be important for invasion and molting. For example, filarial cathepsin L-like proteases (Bm-CPL-1,-4 and -5) are believed to be important for larval migration and molting [39-41]. L3i express a protease (BMC12240), which is analogous to a matrix metalloprotease in the canine hookworm (*Ac-mtp-1*) that is highly expressed in non-activated AcL3 and involved in skin penetration [17,42]. Thus, our results support the concept that proteases involved in tissue migration are over-expressed in helminth stages that infect mammalian hosts [43].

The L3i gene set also includes genes that are believed to be involved in pathogenesis, parasitism and stress resistance. For example, the highly expressed *Bm-val-1 *encodes a venom allergen-like protein that is related to genes in *A. caninum *that encode *Ancylostoma *secreted-proteins (ASPs) [44]. ASPs belong to the pathogenesis-related proteins superfamily (PRPs) [45]; they are associated with the transition to parasitism in hookworms and highly expressed in non-activated AcL3 [16,17,46]. It is interesting that the *O. volvolus *homologue of *Bm-val-1 *(Ov-ASP-1) has been reported to induce anti-L3 immunity in an animal model [47].

The L3i gene set also includes a unique small heat shock protein (sHSP) of *B. malayi *that was represented by two ESTs on the array (BMC02059 and TC3642). This gene was highly expressed in L3i relative to L3c, and its over-expression was confirmed by qRT-PCR (data not shown). This sHSP is developmentally regulated and inducible in *B. malayi *[48]. Small heat shock proteins are among the most highly expressed dauer-specific transcripts in *C. elegans*, and they have a demonstrated role in longevity and stress resistance [49-51].

The last two categories of proteins highly expressed in L3i were surface or cuticular proteins and highly immunogenic proteins. Genes encoding cuticular collagens accounted for 19% of L3i up-regulated known genes (47/249). Their dominant presence is understandable, as the nematode cuticle protects the worms from environmental stress. Cuticular collagens are also needed for the development of the next stage of the parasite (L4) after invasion. *C. elegans *homologs of these genes have RNAi phenotypes of developmental delay such as larval arrest (Lva), larval lethal (Lvl) (BMC0572), and slow growth (Gro) (BMC12441) [See additional file 6]. Collagen genes are over-expressed just before molting in *C. elegans *[51]. The L3i gene set also contains a highly expressed ground-like family member (*grl-4*) [52] (4083.m00057). *Grl-4 *is believed to function during the dauer-to-L4 molt in *C. elegans *[51]. It would be interesting to test whether this gene is also required for molting in filarial worms. The highly immunogenic proteins, such as Bm-VAL, intermediate filament, and microfilaria surface-associated proteins may be targets of protective immunity [28,53]. Filarial SPX-1 and Gln-rich protein have been used as diagnostic antigens [54,55]. These proteins have also been suggested as targets of protective immunity to *Brugia *and *Ascaris *[56,57]. *Brugia *larval allergen (gene 14323.m00082) was differentially recognized by IgE antibody from putatively immune endemic normal humans; this protein induces specific IgE antibody responses in animals (authors' unpublished data).

### The L3ir gene set contains irradiation responsive (IR) genes and highly immunogenic genes that are also up-regulated in L3i

The L3ir gene set includes genes involved in DNA repair and cuticle regeneration after radiation. Genes reported to be IR in other systems were also up-regulated in L3ir as shown in Table 5. These included cytidine deaminase, uridine phosphorylase, aquaporin and finger proteins [58-60]. Notably, various collagen gene families were also over-represented, and these accounted for more than 30% of the total and 50% of L3ir known genes, respectively. Collagen is a principle component of the nematode cuticle that is secreted by the underlying hypodermis, and collagen synthesis is often increased after radiation injury in animals [61,62]. The nematode cuticle protects the parasite from environmental insults, and it may be an important target of immune responses [63,64]. The collagen gene *Ovcol-1 *of *O. volvulus*, a homologue of the *B. malayi *collagen gene *Bmcol*-2 (BMC12441, TC2798) up-regulated in L3ir, is preferentially recognized by immunoglobulin G3 (IgG3) from putatively immune individuals [65]. Cuticular collagens of nematodes are very different from vertebrate collagens, and they are potential targets of protective immune responses [66]. In addition to collagens, the L3ir gene set contains highly immunogenic secretory and membrane proteins; most of these are also present in the L3i gene set. It is interesting that these genes were more highly expressed in L3ir (which induce protective immunity) than in L3c (which do not). It is possible that the enhanced immunogenicity of L3ir is partially due to their persistent high level expression of L3i proteins that are normally down-regulated shortly after L3 enter the mammalian host. This could prolong and enhance priming of the host immune system to critical protective antigens.

### The L3c gene set contains genes that are involved in growth and molting

In contrast to L3i and L3ir, most of the known L3c up-regulated genes were involved in protein expression, reflecting increased protein synthesis during culture (Table 6). Genes encoding various ribosomal proteins accounted for 25% of known L3c genes (38/154). It is interesting that some of these genes have *C. elegans *homologues involved in molting as shown in Table 7[67]. *C. elegans *homologues of those genes have severe embryonic lethal and developmental delay phenotypes such as sterile and larval arrest (13787.m00042 and BMC00239) [See additional file 7]. Our data support the idea that molting requires a burst of biosynthetic activity, presumably to make components for the new cuticle and for growth [67,68]. Other over-represented categories were genes involved in cell cycle and phagocytosis and genes involved in fiber growth. Many of the L3c up-regulated genes are homologues of genes that are also up-regulated in activated (post infection) hookworm L3. These include genes that encode heat shock proteins, tubulin, and rRNA [16,17]. In addition, two oligos matched to small heat shock proteins (sHSPs) (BMC03771 and 15334.m00009) were up-regulated, and this is consistent with previous report [2].

**Table 7:** L3c genes for protein synthesis with *C. elegans *homologs identified in a molting screen*

**Gene**	**Description**	**Oligo IDs**
*rps-10*	Ribosomal protein S10	BMC10905, BMC12256, TC3146, BMC12257, BMC00239, BMC04758
*rps-3*	Ribosomal protein S3, C-terminal	BMC00419, 13787.m00042, BMC06743, BMC12302
*rpl-23*	Ribosomal protein L23e	BMC00275, 14980.m02840, BMC11689
*rpl-18*	Ribosomal L18ae protein	BMC00864, H98325
*rpl-32*	Ribosomal protein L32e	AA841361
*rpl-15*	Ribosomal protein L15e	BMC00151
*rps-11*	ribosomal protein S11	TC2943

*See reference [67]

### Overview of pathways and GO analysis

KEGG and InterPro analyses were performed to functionally classify and categorize genes up-regulated in each L3 type. The complete KEGG pathways identified for each gene set are listed in additional file 8. Significant protein domain matches for each gene set (*P *< 0.05) are shown in additional file 9. The GO associations for whole L3 gene set are available online through the Amigo viewer . Differences in pathways and GO terms inferred by transcriptional expression profiling were observed among different L3 types. It is important to note that the RNA levels do not correlate with protein level in all cases; therefore, such differences are needed to be confirmed using appropriate methods.

### Altered pathways and significant GO terms in L3i gene set

**Figure 2:**
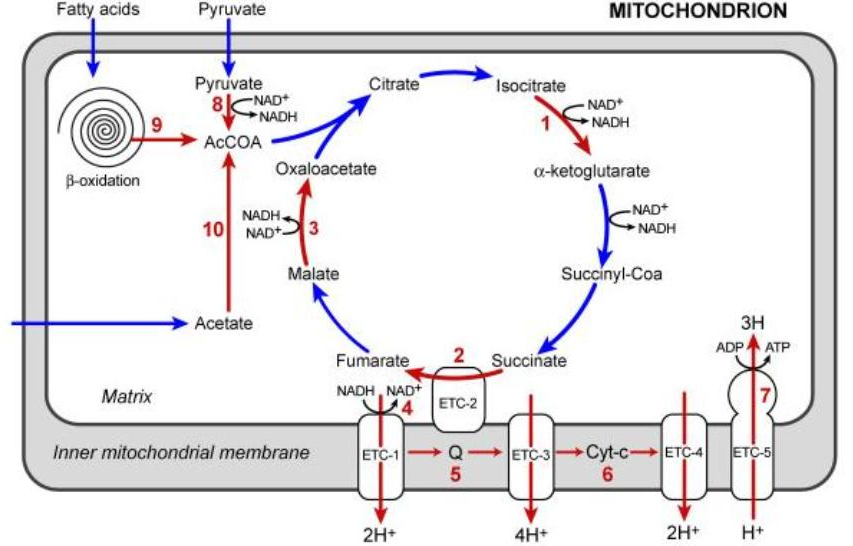
**Overview of the pathways involved in energy generation in BmL3i**. The red arrows and numbers indicate increased levels of enzymatic activity as inferred by fold changes in gene expression. ETC: electron transport chain; Q: electron carrier. The numbers in parentheses are fold changes: 1, isocitrate dehydrogenase-EC 1.1.1.42: 14990.m07912 (3 fold); 2, succinate-ubiquinone oxidoreductase – EC 1.3.5.1: BMC09915 (3 fold)and WB-contig_1179 (4.3); 3, malate dehydrogenase – EC 1.1.1.37: BMC05093 (3 fold), BMC11994 (4 fold); 4, NADH dehydrogenase (ubiquinone): – EC1.6.5.3: there are four elements encoding the enzyme – BMC07652 (3.3 fold), TC3428 (2.9 fold), BMC04790 (4 fold) and 14062.m00071(2.6 fold); 5, Ubiquinol – cytochrome-c reductase or Cytochrome bc1 complex – EC 1.10.2.2: WB-contig_1179 (4.3); 6, Cytochrome c oxidase-EC 1.9.3.1: BMC06398 (2.7); 7, ATP synthase-EC 3.6.3.14: 14980.m02712 (3.4), 14982.m02237 (3.3) and WB-contig_234 (2); 8, Pyruvate dehydrogenase complexes-EC 1.2.4.1: BMC08144 (4.3) and WB-contig_1351 (5.4); 9, Acetyl-CoA C-acyltransferase-EC 2.3.1.16: TC3583 (6.2); 10, Acetyl-coenzyme A synthetase-EC 6.2.1.1: BMC03124 (2.6).

KEGG pathways that were significantly enriched in the L3i gene set are shown in Table 8. An overview of the main up-regulated pathways for energy metabolism in L3i is presented in Figure 2. Developmentally arrested and non-feeding infective L3 were expected to have low energy production. We were surprised to see that the most enriched pathway mapped in the L3i gene set was energy metabolism (oxidative phosphorylation (ko00190)). This suggests that energy production and consumption might be high in L3i. High energy consumption might be needed in part to detoxify endogenous metabolic products or host proteins. For example, the detoxification reactions catalyzed by GSTs and dehydrogenases/reductases are metabolically costly [35]. Energy is also required for invasion as filarial L3 actively migrate immediately after entering the host through subcutaneous tissues en route to lymphatic vessels [69]. However, additional studies will be needed to test these hypotheses. The microarray data show that BmL3i probably use oxidative phosphorylation and the TCA cycle to produce and store energy through aerobic cellular respiration in the form of ATP as shown in Figure 2. All of the enzymes needed for electron transport in the oxidative phosphorylation pathway are up-regulated in L3i. This explains the association of the significant GO term "mitochondria" with these enzymes [See additional file 10]. This also explains high expression of enzymes to detoxify ROS such as superoxide dismutase and catalase (Table 4) in L3i, since complexes I and III of the respiratory chain generate these ROS [36]. NADH and succinate needed for ATP production can be generated in the citric acid cycle (TCA, Ko00020). Genes encoding three TCA enzymes (malate dehydrogenase, succinate dehydrogenase, and isocitrate dehydrogenase) were over-expressed by a factor of 4 in L3i relative to L3c. Acetyl-CoA, the first molecule entering the TCA, is produced through fatty acid β-oxidation; acetyl-CoA C-acyltransferase (K00632, E2.3.1.16, fadA), the enzyme catalyzing the final step of β-oxidation, was highly expressed in L3i [See additional file 8]. This is consistent with the finding in dauer stage of *C. elegans *[51,70]. This suggests that the metabolism of non-feeding BmL3 is adapted to utilize internal energy reserves, predominantly fatty acids. However, there may be the alternate methods to produce Acetyl-CoA in BmL3. For example, Acetyl-CoA might be produced through the pyruvate dehydrogenase complex, as genes encoding two enzymes in the complex were up-regulated; Acetyl-CoA also could be formed by acetate and coenzyme A, as acetyl-coenzyme A synthetase (k01895, E 6.2.1.1) was also over-expressed in L3i. We also found up-regulation of genes encoding enzymes for pyruvate metabolism (ko00620) and gluconeogenesis/glycolysis (ko00010) in L3i including phosphoenolpyruvate carboxykinase (PEPCK) and triose-phosphate isomerase [See additional file 11]. This is also consistent with results reported for dauer larvae in *C. elegans *[[35,49], and [51]].

**Table 8:** Significant KEGG pathways in the L3i gene set

**Pathway**	**ko**	**P_value**	**Metabolism**
**Oxidative phosphorylation**	ko00190	0.0003	Energy Metabolism
**Citrate cycle (TCA cycle)**	ko00020	0.0045	Carbohydrate Metabolism
**Antigen processing and presentation**	ko04612	0.0114	Immune System

GO analysis indicated that genes up-regulated in L3i were significantly involved in four biological processes (namely generation of precursor metabolites and energy GO:0006091; establishment of localization GO:0051234; proteolysis GO:0006508 and coenzyme metabolic process GO:0006732), and associated with three molecular functions (structural constituent of cuticle GO: 0042302; catalytic activity GO: 0003824; and enzyme regulator activity GO: 0030234) [See additional file 10]. This is consistent with the results of the KEGG analysis. BmL3 generate metabolites and energy by oxidative phosphorylation (GO:0006119). Coordinately, genes involved in the molecular function "electron carrier activity" (GO:0009055) are over-expressed in L3i. Most of the genes involved in the molecular function "structural constituent of cuticle" (GO: 0042302) encoded collagens (cuticular collagen 34 and 14 and 2). Interestingly, these collagens were also mapped into the biological process "phosphate transport" (GO: 0006817), and some of these genes were nematode – (BMC09088) or filarial-specific (BMC06834, TC2884). *C. elegans *homologs of up-regulated collagens in L3i had RNAi phenotypes such as embryonic lethal (Emb, BMC05026; BMC12506), developmental delay, and larval lethal (Lvl) (BMC05172) [See additional file 7]. These *B. malayi *collagens merit further study as potential vaccine targets.

Several enzyme activities such as oxidoreductase, hydrolase, and kinases were obviously increased in L3i. These molecular functions were well related to parasite survival (oxidoreductase), parasite invasion and development (hydrolase), and energy production (kinase). The increased enzyme regulator activity (GO:0030234) was mainly for enzyme inhibitor activity (GO: 0004857), especially increased activities of serine-type endopeptidase inhibitor (serpin) and a cysteine protease inhibitor (cystatin). In addition, the products of 14965.m00429 and BMC02136, up-regulated in L3i, were mitochondrial ATPase inhibitors (ATP family protein) associated with down-regulation of nucleotide metabolic processes (GO:0045980, biological process). These molecules might contribute to developmental arrest of L3i in mosquitoes through negative regulation of nucleotide metabolism. GO analysis identified the mitochondrion (GO:0005739) and extracellular region (GO:0005576) as significant cellular localizations for L3i up-regulated genes.

### Altered pathways and GO terms in L3ir and L3c

The same approach was followed to determine functional assignments for the L3ir and L3c gene sets. To our surprise, L3ir may rely heavily on fatty acids for energy during in vitro culture as shown in Table 9. Two genes encoding enzymes involved in fatty acid β-oxidation, namely acetyl-CoA C-acyltransferase (TC3583, 3 fold) and carnitine O-palmitoyltransferase (BMC04165, 2.3 fold; Pub_Locus Bm1_02015, K00636, EC2.3.1.21), were over- expressed in L3ir relative to L3c [See additional file 6]. In addition, genes encoding cellular membrane proteins (BMC02613, 3 fold; 13888.m00008, 4 fold), a family of transmembrane channels called aquaporins (AQPs), were also over-expressed in L3ir relative to L3c; these genes mapped into the "pores ion channels" KEGG pathway (K02440) that facilitates glycerol uptake [71]. Pathways involved in amino acid degradation were also significantly enriched in L3ir. The amino acids include both ketogenic (leucine, lysine and isoleucine) and glucogenic (histidine and cysteine) amino acids. The gene encoding dihydrolipoamide branched chain transacylase (DBT, k00680, E E2.3.1.-) represented by two oligos elements on the array (BMC03018; 14977.m05029, Pub_Locus-Bm1_43910) was also over-expressed in L3ir relative to L3c (3 fold). DBT is involved in the metabolism of amino acids (ko00280, ko00340, ko00310) other than cysteine (ko00272) and also involved in pathways "xenobiotics biodegradation and metabolism" (ko00642, ko00624 and ko00632) and "biosynthesis of secondary metabolites" (ko00903 and ko00960). It is unlikely that all of these pathways are altered in L3ir as a result of up-regulation of DBT. However, there are good reasons to believe that some of the pathways linked to DBT are altered in L3ir. For example, the biosynthesis of many classes of secondary metabolites in plants can be induced by stress or environmental changes [72-74]. In addition, exposure to UV radiation induces the biosynthesis of UV-absorbing compounds such as secondary metabolites (flavonoids) in plants [75]. Cysteine has a crucial role in inducible, endogenous detoxication mechanisms in the body [76]. Taken together, stress responses or IR seem to be appropriately altered in L3ir.

**Table 9:** Significant KEGG pathways identified in the L3ir gene set

**Pathway**	**KO**	**P_value**	**Metabolism**
**Cysteine metabolism**	ko00272	0.0053	Amino Acid Metabolism
**Valine, leucine and isoleucine degradation**	ko00280	0.0053	Amino Acid Metabolism
**Propanoate metabolism**	ko00640	0.0187	Carbohydrate Metabolism
**Alkaloid biosynthesis II**	ko00960	0.0316	Biosynthesis of Secondary Metabolites
**Glycerophospholipid metabolism**	ko00564	0.0316	Lipid Metabolism
**Histidine metabolism**	ko00340	0.0316	Amino Acid Metabolism
**Benzoate degradation via CoA ligation**	ko00632	0.0316	Xenobiotics Biodegradation and Metabolism
**1- and 2-Methylnaphthalene degradation**	ko00624	0.0316	Xenobiotics Biodegradation and Metabolism
**Pores ion channels**		0.0316	Membrane Transport
**Lysine degradation**	ko00310	0.0316	Amino Acid Metabolism
**Fatty acid metabolism**	ko00071	0.0316	Lipid Metabolism
**Limonene and pinene degradation**	ko00903	0.0316	Biosynthesis of Secondary Metabolites
**Ethylbenzene degradation**	ko00642	0.0316	Xenobiotics Biodegradation and Metabolism

The statistically enriched gene ontology list for L3ir genes shared some features with the L3i list [See additional file 12]. However, most of the L3ir up-regulated genes were located in cytoplasm (GO:0005737) in contrast to extracellular region (GO:0005576) for L3i genes. L3ir genes had fewer significant GO terms than L3i or L3c. GO terms related to catalytic activity and enzyme regulatory activity were not enriched in the L3ir gene set.

The only KEGG pathway mapped in the L3c gene set was "ribosome for protein translation". In contrast, L3c had a long list of GO terms [See additional file 13]. However, most of the up-regulated genes were involved in biological processes of protein synthesis and growth such as translation (GO:0006412) and cell development (GO:0048468). Coordinately, the molecular functions related to these biological events were also enriched such as "structural constituent of ribosome" (GO: 0003735), "binding activities of nucleic acid binding" (GO:0003676), and "nucleotide binding" (GO:0000166)). In contrast to L3i, most genes up-regulated in L3c appear to be intracellular (GO:0005622) in various compartments such as organelle (GO:0044446) and microtubule cytoskeleton (GO:0015630).

### Additional comparisons of BmL3 gene expression results with those reported for *C. elegans *and hookworm larvae

We compared gene expression data from BmL3 with data from infective larvae of *A. caninum *(Ac) and L3 dauer larvae of *C. elegans*. Datu et al identified 602 mRNAs differentially transcribed between non-activated- and activated-Ac L3 by serum [17]. We compared transcription profiles of the non-activated AcL3 and mosquito derived BmL3 (L3i). The genes coding cytochrome C oxidase, genes encoding PRPs family member ASP, and alpha-crystallin domain containing proteins (small heat shock proteins except *hsp-12.6*) were up-regulated in both non-activated AcL3 and BmL3i. However, genes encoding catalytic proteases such as cysteine, serine and metalloprotease were up-regulated in BmL3i relative to BmL3c, which contrasts with finding that these genes were up-regulated in activated AcL3 relative to non-activated AcL3. In addition, the lack of similarity between activated AcL3 and BmL3c was observed in gene-by-gene comparisons. For example, PRPs and cysteine protease were up-regulated in activated AcL3 but not in BmL3c. Also BmL3i actively expressed genes involved in invasion and immune evasion, while genes that encode such PRPs were activation-associated in AcL3. The biological significance of these differences remains to be determined. However, it is important to note that AcL3 are free living organisms while BmL3 are parasitic in mosquitoes.

SAGE and microarray studies have identified gene expression profiles associated with dauer arrest and exit in *C. elegans *[49,51]. A total of 1,984 genes identified as dauer exit-specific genes had been further divided into five subclasses: dauer-enriched, transient, early climbing, and late. We compared BmL3 expression data to the dauer exit gene lists, and concordant functional classes are shown in Table 10. Genes involved in stress resistance and lipid metabolism was up-regulated in BmL3i and also in the dauer-enriched subclass. Other BmL3i up-regulated genes were identified in other subclasses especially the "late climbing group". Of course, there were also important differences between BmL3i and dauer exit gene lists. For example, the dauer-enriched gene encoding cytochrome P450 linked to dauer formation [77,78] was not up-regulated in *B. malayi *L3i despite the presence of eight elements on the array annotated as cytochrome P450. Furthermore, *Ce-hsp-12.6 *(designated DAF-16) encodes a Forkhead transcription factor and is a member of the transient gene class [79]. Daf-16 promotes dauer formation and functions downstream of the daf-2/insulin-like receptor signal transduction pathway that regulates dauer development and longevity. There are 6 ESTs annotated as *B. malayi *homologs of small heat shock 12.6 on the array, but none was up-regulated in L3i. Interestingly, the transcription patterns for these two genes are similar in AcL3 and *C. elegans *dauer larvae [16,17]. This suggests that non-activated AcL3 may be more similar to *C. elegans *dauer than BmL3. Again, this may be related to the fact that both AcL3 and *C. elegans *dauer are free-living nematode larvae while BmL3i are parasitic in mosquitoes.

**Table 10:** Overlap of functional classes in L3i genes and *C. elegans *dauer exit-specific genes*

Functional class*	**L3i genes**	***C. elegans *dauer-recovery**				
		**Dauer-enriched**	**Transient**	**Early**	**Climbing**	**Late**
**Lipid metabolism genes**	yes	yes				
**stress related: small HSP, sod**	yes	yes				
**Transporters**	yes		yes	yes		
**TCA**	yes				yes	
**Fatty acid oxidation**	yes				yes	
**Energy generation genes**	yes				yes	
**Protease**	yes					yes
**Patched-like/NPC**	yes				yes	
**Basement membrane**	yes				yes	yes
**Collagen**	yes					

*See reference [51]

### Consistency with prior studies and confirmation by real-time qRT-PCR

The fact that the findings from this study are consistent with previous reports tends to validate our gene expression profiles. For example, prior studies have shown those BmL3i genes such as cathepsin L-like proteases-5 (*Bm*-*cpl-5*), serine protease inhibitor-1 (*Bm-spn-1*), and several *Bm-alt family *genes are highly expressed in BmL3i [41,55,80]. Irradiation responsive genes in the BmL3ir gene set such as cytidine deaminase, uridine phosphorylase and collagens are also up-regulated after irradiation in other organisms [58-60,62]. In addition, expression profiles were confirmed by real-time qRT-PCR for selected gene candidates as shown in additional file 14.

## Conclusion

In summary, our data show that the transition of BmL3 from the mosquito host to culture conditions that mimic the mammalian host is accompanied by important changes in gene expression associated with several biochemical pathways and functions. Major differences involve genes that encode proteins with various functions such as invasion and immune evasion in L3i and growth and development in L3c. Irradiation significantly altered these changes with persistent expression of many L3i genes that are normally down-regulated in culture and increased expression of irradiation response genes. These alterations in gene expression may contribute to the efficacy of L3ir vaccines. Our data support the findings of prior studies that suggested that L3i enriched genes such as *Bm-CPI-2 *and *Bm-ALT-2 *may be involved in establishment of infection and immune evasion. In addition, our analysis greatly extends the number of candidate genes involved in such roles. L3i genes encode proteins newly identified in the secretome of *Brugia *adult worms [81] such as transthyretin-like family proteins (TLP) (TC3177), triose phosphate isomerase (TPI) (BMC11888) and galectin (BMC04762 and BMC12477), each of which fully warrant further investigation. Many of the genes up-regulated in the different L3 types have *C. elegans *homologues with severe RNAi phenotypes [See additional file 7] and some have been previously suggested as potential drug targets [See additional file 15] [82]. Altered pathways associated with L3 types provide clues regarding the molecular basis of important aspects of BmL3 biology such as invasion and evasion (for BmL3i), and growth and development (for BmL3c).

Filarial genomics is a work in progress. The *B. malayi *genome contains all of the puzzle pieces. This study represents a step in the long process of assembling these pieces to provide insight into the biology of filarial nematodes.

## Methods

### Parasite materials

BmL3 were isolated by dissection from *Aedes aegypti *mosquitoes that had been fed 14 days earlier on microfilaremic jirds as previously described [83]. Infective larvae were washed three times with RPMI-1640 (GIBCO-BRL, Grand Island, NY and immediately snap frozen on dry ice and stored at -80°C as mosquito derived infective larvae (L3i) as previously described [5]. Parasite cultures were performed as previously described [84]. Briefly, 2000–2500 L3i were incubated in 5 ml complete NI-medium [NCTC 135 (Sigma Chemical Co., St Louis, MO) and IMDM (GIBCO-BRL, Life Technologies, Grand Island, NY) (1:1) containing 2 mM glutamine, 200 U/ml streptomycin, 200 *ug*/ml penicillin, 0.5 ug/ml amphotericin B, and 10% fetal calf serum in 15 ml conical polystyrene tubes (Costar, Cambridge, Mass) at 37°C in a 95% air, 5% CO_2 _atmosphere for two days. Cultured worms (L3c) were recovered after 2 days, frozen immediately, and stored at -80°C. For irradiated L3 (L3ir), L3i were placed in 2 ml of culture medium in a 5 ml polystyrene tube and exposed to a laboratory certified ^137 ^Cesium source (J.L. Shepherd and Associates, San Fernando, CA) at a dose of 75 krad and then cultured for 2 days as described above for L3c.

### RNA isolation and probe preparation

Total RNA from L3i, L3c and L3ir parasites was prepared using TRIzol (GibcoBRL, Life Technologies) as previously described [5]. cDNA was synthesized from 5–7 *ug *total RNA from each sample using 3DNA capture sequence primers (3DNA Array 350 Detection system, Genisphere, Hatfield, PA) and SuperScript II Reverse Transcriptase (Gibco BRL, Gaithersburg, MD) for each probe according to standard protocols. cDNA was concentrated by Microcon YM-100 filter (Millipore) and either used immediately or stored at -80°C. cDNA was synthesized from two different RNA samples from each L3 type. A two-step protocol was used for hybridization (3DNA Array 350 Detection system, Genisphere, Hatfield, PA) as previously described [85]. Each experiment consisted of a pair-wise competitive hybridization of cDNA samples (L3i/L3c and L3ir/L3c) with reciprocal dye-flip replicates. Since biological replicates and dye-flip replicates were tested, a total of four DNA microarrays were used for each comparison of two types of cDNA.

### Microarray fabrication

The BmV2array contains 18,104 elements derived from *B. malayi *(15,412), *Onchocerca volvulus *(*O. volvulus*, 1,016), *Wuchereria bancrofti *(*W. bancrofti*, 872) and *Wolbachia *(*wBm*, 804 genomic elements). The *Brugia *elements represent all *B. malayi *non-redundant gene models annotated in the course of the *B. malayi *genome project  and ESTs from the TIGR Gene Indices (*B. malayi *v. 5.0 http://compbio.dfci.harvard.edu/tgi/tgipage.html) that did not match *B. malayi *gene models (which may represent un-annotated genes). Other elements represented *O. volvulus *and *W. bancrofti *ESTs that differ significantly from known *B. malayi *genes. The array also displays oligos designed on each *wBm *gene model. ArrayOligoselector [86] was used according to default parameters to choose unique 65 mers for each gene model and EST. The oligos were screened to avoid cross-hybridization to non-redundant human, bacterial and viral data nucleotide databases , and synthesized by standard methods by Illumina (San Diego, CA). The oligonucleotides (50 nM in 3× SSC with 0.75 M betaine) were printed in duplicate on MWG Epoxy slides (MWG Bioteche Inc, High Point, NC) by a locally constructed linear servo-arrayer (after the DeRisi model, ).

### Data processing and analysis

Slide scanning and image analysis were performed as described previously (85). Briefly, slides were scanned immediately after hybridization on a ScanArray Express HT Scanner (Perkin Elmer, Boston, MA) to detect Cy3 and Cy5 fluorescence at 543 and 633 nm, respectively. The scanner produces green Cy3 and red Cy5 16-bit TIFF image files and extracts intensities from the scanner images for both dyes. The resultant values were background subtracted and Lowess [86,87] normalized by using GeneSpring version 6.1 software (Silicon Genetics, Redwood City, CA). Twenty percent of the data were used to calculate the Lowess fit at each point. Genes with fold differences ≥ 2 and *P *< 0.01 based on pair-wise comparisons (L3i/L3c, L3c/L3ir) were considered to be differentially expressed. The best *P*-value obtained with high PMT scans and its accompanying arithmetic ratio was reported.

### Annotation and Functional Assignments

A similarity search was performed using WU-TBLASTX [88] with the 18,104 elements on the chip as queries versus the NCBI NR database (downloaded on Nov 14, 2006). Homologies were reported with a probability of 1E-05 or better. To identify cases where *Brugia *homologs in *C. elegans *have been surveyed for knockout phenotypes using RNA interference (RNAi) [18,19,21], Wormpep BLAST matches were cross referenced to a list of 19, 793 *C. elegans *genes with available RNAi information (Version 156). For each *Brugia *sequence, only the highest-scoring *C. elegans *match was considered.

Default parameters for InterProScan v13.1 were used to search against the InterPro database [89]. Gene ontology (GO) was assigned and displayed graphically by AmiGO with default parameters and ontology data released on March 15, 2007 [15]. For each GO term, its enrichment in a subset group (such as the L3i group) was measured over background using a hypergeometric analysis with the *P*-value cutoff e-05. Less informative ontology terms, including those at level 4 or higher for Biological Process or Molecular Function, and those at level 2 or higher for Cellular Component, were removed from the enrichment list.

An *E*-value cut-off of 1.0e^-10 ^reported by WU-BLASTP against Genes Database Release 39.0 from Kyoto Encyclopedia of Genes and Genomes was used for pathway mapping; the top match and all the matches within a range of 30% of the top BLAST score that met the cut-off were accepted as valid KEGG associations [90,91]. A hypergeometric analysis (measuring the coverage of KEGG Orthology or KO gene groups for each KEGG pathway compared to the complete gene set on the V2 chip) was implemented to identify enriched pathways for each gene set [92].

### Validation of transcription by real-time qRT-PCR

Reverse transcription real-time PCR was used for the validation of microarray data as preciously described [85]. The gene coding for histone (BMC12346) was verified and used as endogenous control as previously reported. The sequences of all of the primers used in the real-time PCR are listed in additional file 16.

## Abbreviations

L3i: infective L3; L3ir: irradiated and cultured L3; L3c: cultured L3; KEGG: Kyoto Encyclopedia of Genes and Genomes; qRT-PCR: quantitative reverse transcription polymerase chain reaction; RNAi: RNA interference.

## Authors' contributions

BWL: experimental design, interpretation of results, data analysis, drafted manuscript. ACR: RNA extraction, qRT-PCR, data mining. MM: genome mining, manuscript preparation. YY: genome mining, statistical analysis, manuscript preparation. DS: oligo calling, genome mining. EG: oligo calling, genome mining, manuscript preparation. GJW: experimental design, interpretation of results, manuscript preparation. All authors have read and approved the final manuscript.

## Supplementary Material

Additional file 1 **Summary of BLAST output of BmV2array elements vs. NCBI_NR**. The data provided represent a similarity search of BmV2 array sequences against NCBI non-redundant database (downloaded Nov 14, 2006). Click here for file

Additional file 2 **Complete RNAi information of *C. elegans *homologues of *Brugia *genes on BmV2array**. The *C. elegans *homologues of *Brugia *genes on the BmV2array were found to have various RNAi phenotypes. Click here for file

Additional file 3 **Summary of protein domain matches for BmV2array elements**. The protein domain matches of BmV2array against InterProScan v13.1 database. Click here for file

Additional file 4 **Summary of KEGG analysis of BmV2array**. The top match of pathway mapping for BmV2array sequences. Click here for file

Additional file 5 **Top 20 protein domain matches for BmV2array elements**. The data provided represent top 20 protein domains matched in BmV2array sequences. Click here for file

Additional file 6 **Up-regulated genes in the L3 type**. The up-regulated genes in pair-wise comparison of L3i vs L3c and L3ir vs L3c. Click here for file

Additional file 7 **RNAi phenotypes of *C. elegans *homologues in L3 the gene sets**. The *C. elegans *homologues of *Brugia *genes up-regulated in L3 (L3i, L3ir and L3c) were found to have various RNAi phenotypes. Click here for file

Additional file 8 **Complete KEGG analysis in the L3 gene set**. The KEGG pathway mapped in the L3 gene set. Click here for file

Additional file 9 **Significant protein domains in each L3 gene set**. The most significant protein domains matched in each L3 gene set. Click here for file

Additional file 10 **Significant GO terms in the L3i gene set**. The statistically significant Gene Ontology terms in the L3i gene set. Click here for file

Additional file 11 **L3i genes involved in gluconeogenesis**. The genes up-regulated in L3i were involved in gluconeogenesis. Click here for file

Additional file 12 **Significant GO terms in the L3ir gene set**. The statistically significant Gene Ontology terms were mapped in the L3ir gene set. Click here for file

Additional file 13 **Significant GO terms in the L3c gene set**. The statistically significant Gene Ontology terms in the L3c gene set. Click here for file

Additional file 14 **Up-regulated genes confirmed by qRT-PCR**. Genes up-regulated by microarray were confirmed by real-time qRT-PCR. Click here for file

Additional file 15 **Potential drug targets in each L3 gene set**. The potential drug targets were identified in each L3 gene set. Click here for file

Additional file 16 **Primers of selected genes confirmed by real-time qRT-PCR**. The primers used in real-time qRT-PCR for the confirmation. Click here for file
