# Supplementary material for: Transcriptomes and pathways associated with infectivity, survival and immunogenicity in Brugia malayi L3
Source: BMC Genomics. 2009 Jun 15;10:267. doi: 10.1186/1471-2164-10-267 (PMC2708187; doi:10.1186/1471-2164-10-267)
Supplement: Additional file 5 — Top 20 protein domain matches for BmV2array elements. The data provided represent top 20 protein domains matched in BmV2array sequences. [file 1471-2164-10-267-S5.docx]

Table S5. Top 20 most abundant protein domain matches on the BmV2array

| **IPR** | **Description** | **# of elements** |
| --- | --- | --- |
| IPR011009 | Protein kinase-like | 290 |
| IPR007087 | Zinc finger, C2H2-type | 280 |
| IPR000719 | Protein kinase, core | 275 |
| IPR002290 | Serine/threonine protein kinase | 182 |
| IPR012677 | Nucleotide-binding, alpha-beta plait | 158 |
| IPR001680 | WD40 repeat | 153 |
| IPR001245 | Tyrosine protein kinase | 150 |
| IPR011046 | WD40-like | 140 |
| IPR008160 | Collagen triple helix repeat | 138 |
| IPR000504 | RNA recognition motif, RNP-1 | 134 |
| IPR013032 | EGF-like region | 132 |
| IPR012336 | Thioredoxin-like fold | 120 |
| IPR008271 | Serine/threonine protein kinase, active site | 114 |
| IPR000583 | Glutamine amidotransferase, class-II | 111 |
| IPR012335 | Thioredoxin fold | 107 |
| IPR003006 | Immunoglobulin/major histocompatibility complex motif | 106 |
| IPR002048 | Calcium-binding EF-hand | 103 |
| IPR013783 | Immunoglobulin-like fold | 99 |
| IPR011992 | EF-Hand type | 99 |
| IPR010916 | TonB box, N-terminal | 96 |
